# Silencing of lncRNA MALAT1 facilitates erastin-induced ferroptosis in endometriosis through miR-145-5p/MUC1 signaling

**DOI:** 10.1038/s41420-022-00975-w

**Published:** 2022-04-11

**Authors:** Zongwen Liang, Qiong Wu, Honglin Wang, Jiahuan Tan, Han Wang, Yanling Gou, Yingying Cao, Zhi Li, Zongfeng Zhang

**Affiliations:** Department of Obstetrics and Gynecology, Second Affiliated Hospital of Harbin Medical University, 150086 Harbin, China

**Keywords:** Cell death, Reproductive disorders

## Abstract

Endometriosis is a chronic disorder characterized by the implantation of endometrial glands and stroma outside the uterus. However, the pathogenesis of endometriosis is still unclear. To date, there is no fully effective treatment without trauma because of various side effects. Recent data suggest that ferroptosis is a novel recognized form of nonapoptosis-regulated cell death characterized by iron-dependent and lethal lipid peroxidation accumulation, showing great promise in the treatment of many diseases. In the present study, we verified that erastin induced ferroptosis in ectopic endometrial stromal cells (EESCs). Furthermore, we found that the expression of metastasis-associated lung adenocarcinoma transcript 1 (MALAT1) was decreased during erastin-induced ferroptosis. Knockdown of MALAT1 significantly aggravated the inhibition of cell viability and increased intracellular iron, Liperfluo, and MDA levels in EESCs upon erastin treatment. Mechanistically, we demonstrated that MALAT1 served as a competing endogenous RNA of miR-145-5p to regulate the expression of MUC1, a suppressor of ferroptosis. MALAT1 knockdown-mediated ferroptotic cell death and MUC1 downregulation could be abrogated by inhibition of miR-145-5p. In addition, miR-145-5p inhibition-mediated ferroptotic cell death could be abolished by MUC1 knockdown. Furthermore, erastin-induced ferroptosis shrunk endometriotic lesions via the MALAT1/miR-145-5p/MUC1 axis in vivo. Taken together, our data indicate that knockdown of MALAT1 facilitates ferroptosis upon erastin treatment via miR-145-5p/MUC1 signaling. The synergistic effect of MALAT1 knockdown and erastin induction in ferroptosis may be a new therapeutic strategy for endometriosis.

## Introduction

Endometriosis is a chronic disease characterized by the implantation of endometrial tissue (gland and stroma) outside the uterine cavity, mainly on the ovaries, pelvic peritoneum, and sacral ligaments [1]. It affects up to 10% of reproductive-age women [2], and that number increases to 30–50% in symptomatic premenopausal women [3]. However, there is currently no fully effective treatment because of various side effects. Therefore, it is urgent to find a satisfactory treatment for endometriosis.

Ferroptosis is a new form of programmed cell death characterized by iron-dependent and lethal lipid peroxidation accumulation and is distinguished from apoptosis, necroptosis, and other reported forms of cell death [4]. As described below, many genes have been found to be involved in ferroptosis by modulating lipid peroxidation, and amino acid metabolism. For example, acyl-CoA synthetase long-chain family member-4 (ACSL4) promotes the biosynthesis of polyunsaturated fatty acid (PUFA)-containing phospholipids, which are the main substrates of lipid peroxidation in ferroptosis [5]. Solute carrier family 7 member 11 (SLC7A11), also known as xCT, is a component of the cysteine/glutamate transporter that imports extracellular cystine and exports intracellular glutamate. Blocking SLC7A11 transporter activity leads to glutathione depletion and inactivation of phospholipid hydroperoxide glutathione peroxidase 4 (GPX4) and further induces ferroptosis [6, 7]. In addition, mucin 1 (MUC1) is a ferroptosis-related gene that forms a complex with xCT and protects against treatment with erastin [8, 9].

Erastin, a small molecule inducer of ferroptosis, inhibits system xc- and triggers ferroptosis by leading to glutathione depletion and inactivation of GPX4 [10]. Previous studies have shown that many tumor cells are sensitive to erastin, which can be used as a new therapeutic strategy for many diseases [11]. However, although Li et al. suggested that ferroportin (FPN) is a negative factor in erastin-induced ferroptosis in EESCs [12], the mechanisms underlying ferroptosis in endometriosis have not been identified.

LncRNAs are a type of non-coding RNA of more than 200 nucleotides, with a limited coding capacity [13]. LncRNAs function as competitive endogenous RNAs (ceRNAs) by “sponging” target miRNAs to regulate the expression of downstream mRNAs [14]. Accumulating evidence has confirmed that lncRNAs are involved in ferroptosis processes in different diseases [15, 16]. Recently, long noncoding RNA metastasis-associated lung adenocarcinoma transcript 1 (MALAT1) was demonstrated to be involved in ROS production (a critical event of ferroptosis) [17]. However, whether MALAT1 is involved in ferroptosis in endometriosis remains unclear.

In the present study, we found that MALAT1 was downregulated in erastin-induced ferroptosis in endometriosis. Mechanistically, we found that knockdown of MALAT1 facilitates erastin-induced ferroptosis in EESCs through miR-145-5p/MUC1 signaling. Finally, we further verified that erastin-induced ferroptosis could shrink endometriotic-like lesions through the miR-145-5p/MUC1 axis in vivo.

## Results

### Ferroptosis resistance occurs in endometriosis

**Fig. 1:**
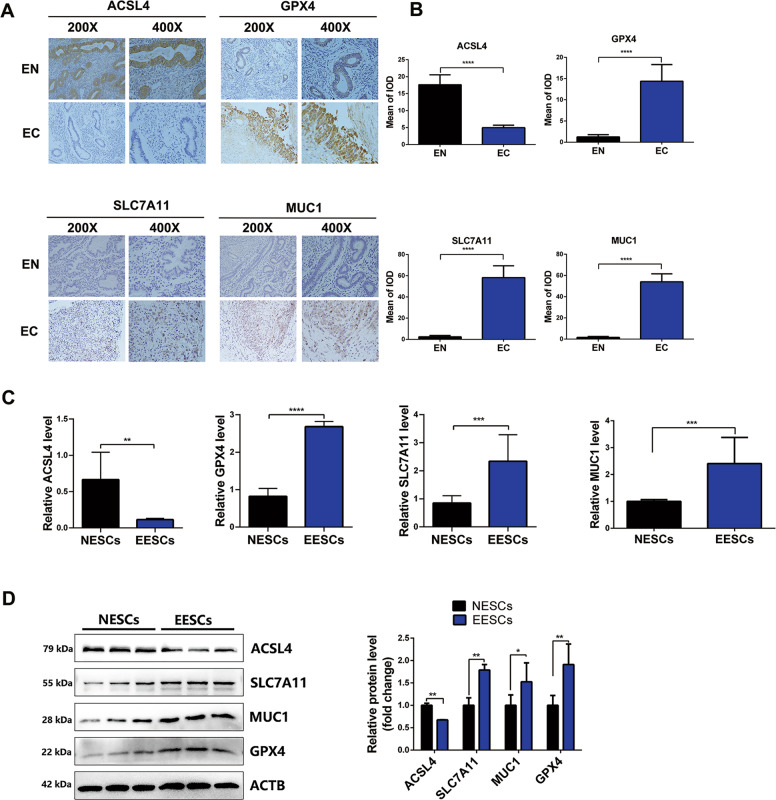
Ferroptosis resistance occurs in endometriosis. **A** Representative images of ferroptosis-related protein (ACSL4, GPX4, SLC7A11, and MUC1) expression in EN and EC tissues were analyzed by immunohistochemical staining. The scale bar = 100 µm for ×200, 50 µm for ×400. **B** Semiquantitative analysis software (ImageJ) was used to measure the mean IOD of the sections. **C** The mRNA levels of ACSL4, GPX4, SLC7A11, and MUC1 in NESCs and EESCs were determined using quantitative RT-PCR (qRT-PCR) analysis. **D** Protein expression of ACSL4, GPX4 SLC7A11, and MUC1 in NESCs and EESCs was determined using western blot analysis. ImageJ was used to calculate the integrated density of the protein bands. All data are shown as the mean ± SD of three independent experiments (*P < 0.05, **P < 0.01, ***P < 0.001, ****P < 0.0001). Statistical significance was calculated using Student’s *t* test. EC ectopic endometrial, EN normal endometrial, NESC normal endometrial stromal cell, EESC ectopic endometrial stromal cell, ACSL4 acyl-CoA synthetase long-chain family member 4, GPX4 glutathione peroxidase 4, SLC7A11 solute carrier family 7 member 11, MUC1 mucin 1, IOD integrated optical density.

To explore ferroptosis status in endometriosis, we performed immunohistochemical staining of ferroptosis-related proteins in human EC tissues and EN tissues. As expected, we found that the expression level of ACSL4 was decreased, while the expression levels of GPX4, SLC7A11, and MUC1 were increased in EC tissues compared to EN tissues (Fig. 1A, B). In addition, the mRNA level of ACSL4 was found to be downregulated in EESCs compared to NESCs. In contrast, the mRNA levels of GPX4, SLC7A11, and MUC1 were upregulated in EESCs compared to NESCs (Fig. 1C). Similarly, the protein level of ACSL4 was decreased, while the protein levels of GPX4, SLC7A11, and MUC1 were increased in EESCs compared to NESCs (Fig. 1D). These results suggested that ferroptosis resistance occurred in endometriosis.

### Erastin induces ferroptosis in EESCs

**Fig. 2:**
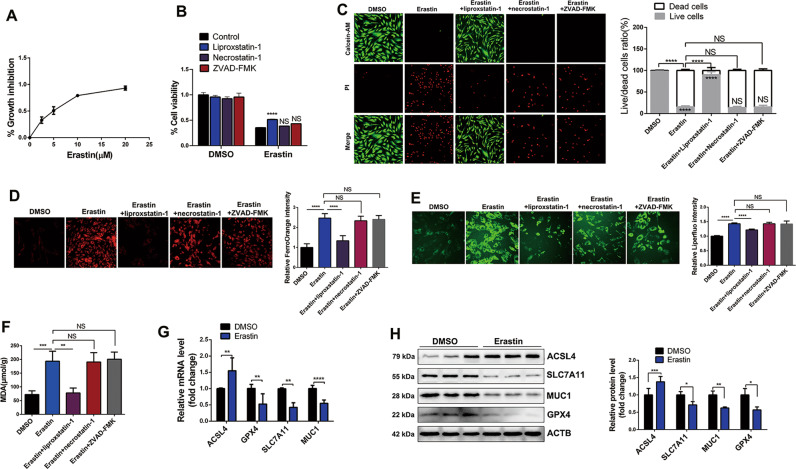
Erastin triggers ferroptosis in endometriosis. **A** EESCs were treated with the indicated concentrations of erastin (2.5, 5, 10, and 20 µM) in 96-well plates for 24 h. CCK-8 assay was used to detect cell viability. **B**–**F** EESCs were treated with erastin (10 μM) with or without the indicated inhibitors (liproxstatin-1, 1 μM; necrostatin-1, 10 µM; ZVAD-FMK, 10 μM) for 24 h. **B** Cell viability was detected by a CCK-8 assay. **C** Living and dead cells were stained with a Calcein-AM/PI Double Stain Kit. Green represents viable cells, red represents dead cells, and the scale bar = 100 μm. Cell viability was expressed as the percentage of viable cells. **D** Cells were treated with 1 μM FerroOrange to detect intracellular Fe^2+^. Intracellular Fe^2+^ visualized by FerroOrange (magnification, ×200). **E** Cells were treated with 5 µM Liperfluo to detect lipid peroxidation. Ferroptosis marker visualized by Liperfluo (magnification, ×200). **F** MDA levels were assayed after the indicated treatment. **G** qRT-PCR was used to detect the mRNA levels of ACSL4, GPX4, SLC7A11, and MUC1 in EESCs after erastin (10 µM) or DMSO treatment for 24 h. **H** Western blot analysis was used to detect the protein levels of ACSL4, GPX4, SLC7A11, and MUC1 in EESCs after erastin (10 µM) or DMSO treatment for 24 h. All data are shown as the mean ± SD of three independent experiments (NS is nonsignificant, *P < 0.05, **P < 0.01, ***P < 0.001, ****P < 0.0001). Statistical significance was calculated using Student’s t test. EESC ectopic endometrial stromal cell, MDA malondialdehyde, ZVAD-FMK benzyloxycarbonyl-Val-Ala-Asp (OMe)-fluoromethylketone.

Erastin is a small molecular chemotherapy drug that induces ferroptosis [18]. To explore the suitable concentration of erastin intervention in endometriosis, we treated EESCs with erastin in a dose-dependent manner ranging from 0 to 20 μM for 24 h. A significant change was observed at approximately 10 µM (Fig. 2A). Hence, 10 µM erastin was used to induce EESCs ferroptosis in the following experiments. Interestingly, we found that erastin-mediated growth inhibition in EESCs was blocked by liproxstatin-1 (a recognized inhibitor of ferroptosis) but not necrostatin-1 (a recognized necroptosis inhibitor) and ZVAD-FMK (a recognized apoptosis inhibitor) using a CCK-8 assay (Fig. 2B). Moreover, calcein-AM-propidium iodide (PI) double staining, a cell permeabilization assay, showed that erastin treatment resulted in a significant reduction in live cells and an increase in dead cells compared with the DMSO group, whereas liproxstatin-1, but not necrostatin-1 and ZVAD-FMK, completely reversed the promoting effect of erastin on cell death (Fig. 2C). Given that redox-active iron accumulation and lipid peroxidation are critical events in ferroptosis, we detected the levels of intracellular Fe^2+^, Liperfluo (a marker of ferroptosis), and MDA (malondialdehyde, the end products of lipid peroxidation) in EESCs treated with erastin. As shown in Fig. 2D–F, intracellular Fe^2+^ accumulation and the levels of Liperfluo and MDA were significantly increased following treatment with erastin. Attractively, liproxstatin-1, but not necrostatin-1 and ZVAD-FMK, completely abolished intracellular Fe^2+^, Liperfluo, and MDA levels in the induction of ferroptosis. Furthermore, erastin-mediated overexpression of ACSL4 at both the RNA and protein levels in EESCs was confirmed by qRT-PCR (Fig. 2G) and western blot (Fig. 2H), respectively. Conversely, the mRNA and protein levels of GPX4, SLC7A11, and MUC1 were found to be downregulated in erastin-treated cells compared to the DMSO group (Fig. 2G, H). Overall, these results indicated that erastin could induce EESCs ferroptosis in vitro.

### Knockdown of MALAT1 facilitates erastin-induced ferroptosis in EESCs

**Fig. 3:**
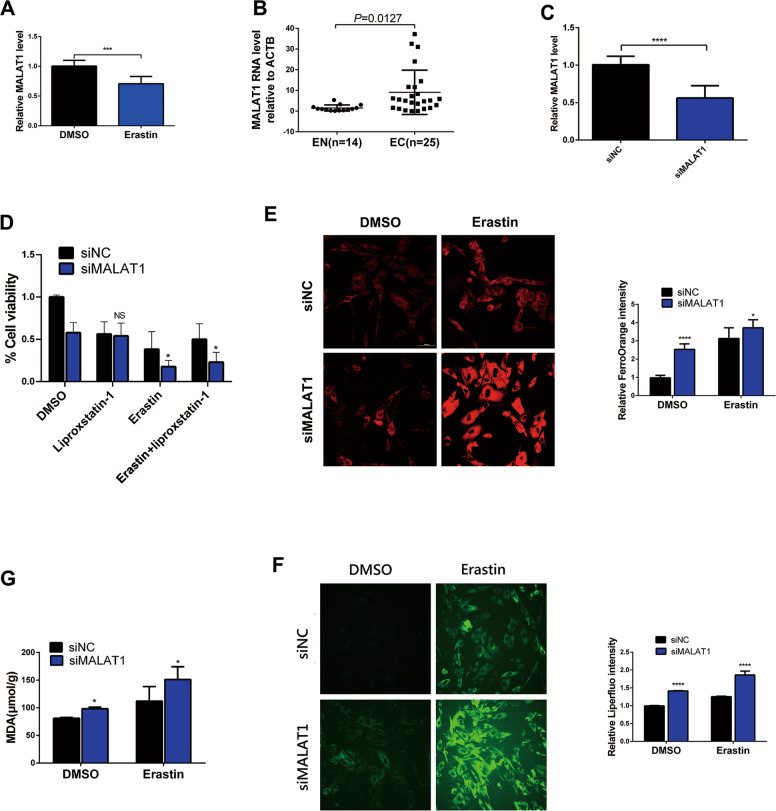
Knockdown of MALAT1 facilitates erastin-induced ferroptosis in EESCs. **A** qRT-PCR analysis was conducted to test the expression level of MALAT1 in EESCs after treatment with erastin (10 µM) or DMSO for 24 h. **B** The mRNA levels of MALAT1 in 25 EC tissues and 14 EN tissues were detected by qRT-PCR. **C** qRT-PCR analysis was performed to detect the efficiency of siRNA-mediated knockdown of MALAT1 in EESCs that were transiently transfected with siMALAT1 (50 nM) or siNC (50 nM) for 24 h. **D** Cell growth was detected by CCK-8 assay upon erastin (10 μM) and/or liproxstatin-1 (1 μM) treatment in MALAT1 knockdown cells. **E**, **F** Cells were treated with 1 µM FerroOrange and 5 µM Liperfluo to detect intracellular Fe^2+^ and lipid peroxidation after MALAT1 knockdown upon erastin (10 μM) treatment. **E** Intracellular Fe^2+^ visualized by FerroOrange (magnification, ×200). **F** Ferroptosis marker visualized by Liperfluo (magnification, ×200). **G** MDA levels were assayed in MALAT1 knockdown cells under erastin (10 μM) treatment. All data are shown as the mean ± SD of three independent experiments (NS is nonsignificant, *P < 0.05, ***P < 0.001, ****P < 0.0001). Statistical significance was calculated using Student’s *t* test. EC ectopic endometrial, EN normal endometrial, EESC ectopic endometrial stromal cell, MALAT1 metastasis-associated lung adenocarcinoma transcript 1, siNC negative control siRNA, siMALAT1 siRNA targeting MALAT1.

LncRNA MALAT1 can protect cells from cytotoxic injury mediated by oxidative stress and lipid peroxidation (a critical event of ferroptosis) [19]. Therefore, we hypothesized that MALAT1 may play an important role in erastin-induced ferroptosis in endometriosis. To test this hypothesis, we performed qRT-PCR analysis and observed that MALAT1 mRNA expression was decreased after erastin treatment (Fig. 3A). Moreover, we selected 25 EC tissue samples and 14 EN tissue samples. The qRT-PCR assay results showed that the expression of MALAT1 was significantly upregulated in EC tissues compared with EN tissues (Fig. 3B). These results showed that the expression of MALAT1 was increased in ferroptosis-resistant EC tissues but was decreased during erastin-induced ferroptosis. In addition, we knocked down MALAT1 expression by transfecting EESCs with MALAT1 siRNA (siMALAT1). The efficiency of siRNA-mediated knockdown of MALAT1 was further confirmed by qRT-PCR analysis (Fig. 3C). Moreover, the results of the CCK-8 assay showed that MALAT1 knockdown aggravated cell growth inhibition upon erastin treatment, which was significantly reversed by the ferroptosis inhibitor liproxstatin-1 (Fig. 3D). Furthermore, MALAT1 knockdown enhanced the levels of intracellular Fe^2+^, Liperfluo, and MDA in EESCs upon erastin treatment (Fig. 3E–G). Collectively, these findings suggest that knockdown of MALAT1 facilitated erastin-induced ferroptosis in EESCs.

### MALAT1 acts as a ceRNA for miR-145-5p

**Fig. 4:**
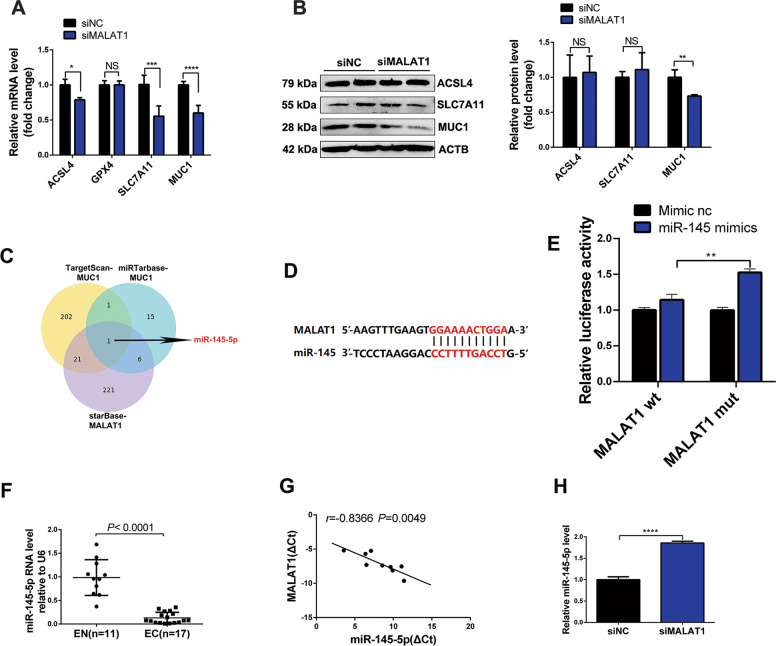
MALAT1 acts as a ceRNA for miR-145-5p. **A** qRT-PCR analysis was performed to detect the mRNA expression of ACSL4, GPX4, SLC7A11, and MUC1 in EESCs transfected with siMALAT1 (50 nM) or siNC (50 nM) for 24 h. **B** Western blot analysis was performed to detect the protein expression of ACSL4, SLC7A11, and MUC1 in EESCs transfected with siMALAT1 (50 nM) or siNC (50 nM) for 48 h. **C** Venn graph of MALAT1-targeted miRNAs predicted by starBase and miRNAs predicted by TargetScan and miRTarBase to target MUC1. **D** The predicted binding sites between miR-145-5p and MALAT1 are shown. **E** Luciferase activities were assessed in 293T cells cotransfected with the MALAT1 mutant (MALAT1 mut) and miR-145-5p mimics or cotransfected with MALAT1 wild type (MALAT1 wt) and miR-145-5p mimics. **F** qRT-PCR analysis was performed to detect the expression of miR-145-5p in 17 EC tissues and 11 EN tissues. **G** The correlation between the expression of MALAT1 and miR-145-5p was analyzed using the Pearson’s test (*r* < 0 denotes a negative correlation). **H** miR-145-5p expression after MALAT1 silencing was determined using qRT-PCR. All data are shown as the mean ± SD of three independent experiments (NS is nonsignificant, *P < 0.05, **P < 0.01, ***P < 0.001, ****P < 0.0001). Statistical significance was calculated using Student’s *t* test. EC ectopic endometrial, EN normal endometrial, EESC ectopic endometrial stromal cell, MALAT1 metastasis-associated lung adenocarcinoma transcript 1, siNC negative control siRNA, siMALAT1 siRNA targeting MALAT1.

Previous studies reported that MALAT1 was localized in cell cytoplasm by RNA fluorescence in situ hybridization (RNA FISH) [20]. Therefore, we hypothesized that MALAT1 acts as a competing endogenous RNA (ceRNA) to regulate downstream genes by sponging common miRNAs, and further regulates ferroptosis in EESCs. We found that only the mRNA and protein levels of MUC1 but not ACSL4, GPX4, and SLC7A11, were significantly decreased by siMALAT1 in EESCs (Fig. 4A, B). To predict the possible common miRNAs between MALAT1 and MUC1, we used three online prediction databases: TargetScan, miRTarBase, and starBase. The Venn diagram suggested that miR-145-5p was the only predicted miRNA across the three prediction tools (Fig. 4C). The putative binding sites between MALAT1 and miR-145-5p were demonstrated by starBase v2.0 (Fig. 4D). To further investigate the interaction between MALAT1 and miR-145-5p, we performed a luciferase reporter assay. The results showed that the luciferase activity was increased in cells cotransfected with MALAT1 mutant and miR-145 mimics compared with cells cotransfected with the MALAT1 wild type and miR-145 mimics (Fig. 4E). In addition, the expression of miR-145-5p was found to be significantly downregulated in EC tissues compared to EN tissues by qRT-PCR (*P* < 0.0001) (Fig. 3F). Pearson’s test showed that there was a negative correlation between the expression of MALAT1 and miR-145-5p in EC tissues (Fig. 4G). Moreover, the level of miR-145-5p was significantly increased in cells transfected with siMALAT1 compared to cells transfected with siRNA negative control (siNC) (Fig. 4H). These results indicated that MALAT1 acted as a ceRNA for miR-145-5p in EESCs.

### MiR-145-5p promoted erastin-induced ferroptosis in EESCs by regulating MUC1

**Fig. 5:**
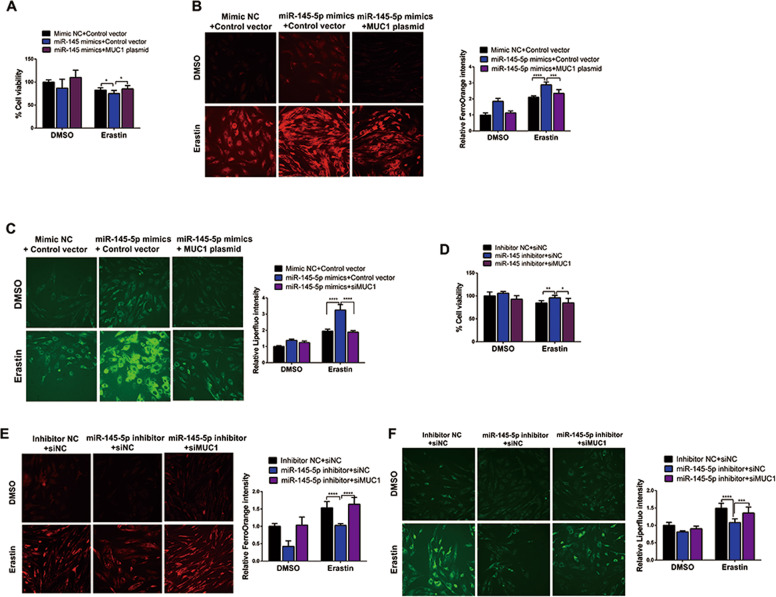
MiR-145-5p promoted erastin-induced ferroptosis in EESCs by regulating MUC1. **A** Cell viability was evaluated in miR-145-5p-overexpressing cells after cotransfection with the MUC1 plasmid. **B**, **C** Intracellular Fe^2+^ and Liperfluo levels were evaluated in miR-145-5p-overexpressing cells after cotransfection with the MUC1 plasmid (magnification, ×200). **D** Cell viability was evaluated in cells with miR-145-5p inhibition after cotransfection with siMUC1. **E**, **F** Intracellular Fe^2+^ and Liperfluo levels were evaluated in miR-145-5p-inhibited cells after cotransfection with siMUC1 (magnification, ×200). All data are shown as the mean ± SD of three independent experiments (NS is nonsignificant, *P < 0.05, **P < 0.01, ***P < 0.001, ****P < 0.0001). Statistical significance was calculated using Student’s *t* test. EESC ectopic endometrial stromal cell, MUC1 mucin 1, siNC negative control siRNA, siMUC1 siRNA targeting MUC1, mimic NC negative control miRNA mimic, inhibitor NC negative control miRNA inhibitor.

MUC1 has been repeatedly reported to be a direct target of miR-145 [21, 22]. To explore whether miR-145-5p promotes erastin-induced ferroptosis in EESCs by regulating MUC1, we performed a CCK-8 assay, and the results suggested that miR-145-5p mimics increased cell growth inhibition, which was reversed by MUC1 overexpression upon erastin treatment (Fig. 5A). In addition, the intracellular Fe^2+^ levels and Liperfluo signal were also greatly rescued by MUC1 overexpression in cells transfected with miR-145-5p mimics upon erastin treatment (Fig. 5B, C). In contrast, cell growth inhibition, intracellular concentrations of Fe^2+^, and Liperfluo signal were reversed by MUC1 knockdown in cells transfected with miR-145-5p inhibitor under erastin treatment (Fig. 5D–F). These data indicated that miR-145-5p promoted erastin-induced ferroptosis in EESCs by regulating MUC1.

### Knockdown of MALAT1 facilitates erastin-induced ferroptosis in EESCs through miR-145-5p/MUC1 signaling

**Fig. 6:**
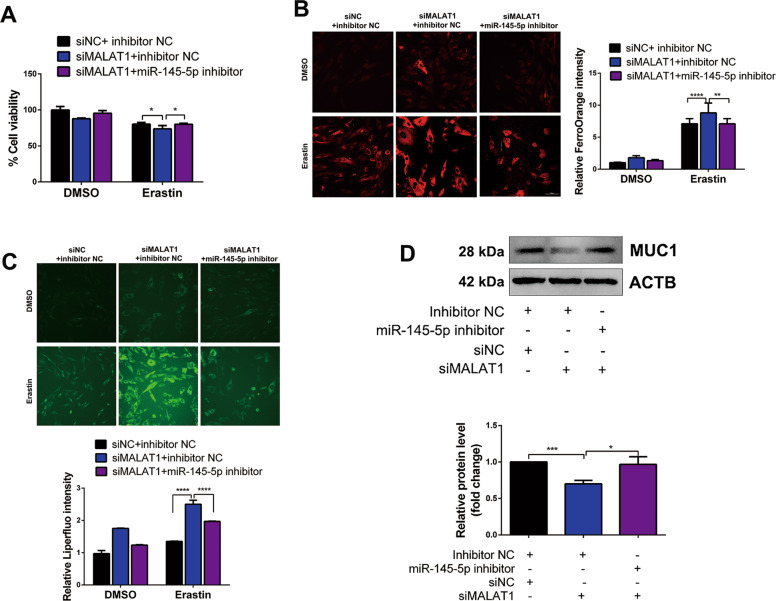
Knockdown of MALAT1 facilitates erastin-induced ferroptosis in EESCs through miR-145-5p/MUC1 signaling. **A** Cell viability was evaluated in MALAT1 knockdown cells after cotransfection with miR-145-5p inhibition. **B**, **C** Intracellular Fe^2+^ and Liperfluo levels were evaluated in MALAT1 knockdown cells after cotransfection with miR-145-5p inhibition. **D** The MUC1 protein level was assessed by western blot in MALAT1 knockdown cells after transfection with miR-145-5p inhibitor. All data are shown as the mean ± SD of three independent experiments (*P < 0.05, **P < 0.01, ***P < 0.001, ****P < 0.0001, Student’s *t* test). EESC ectopic endometrial stromal cell, MALAT1 metastasis-associated lung adenocarcinoma transcript 1, MUC1 mucin 1, siNC negative control siRNA, siMALAT1 siRNA of MALAT1, inhibitor NC negative control miRNA inhibitor.

To explore whether MALAT1 knockdown promotes erastin-induced ferroptosis through miR-145-5p/MUC1 signaling, we performed a CCK-8 assay, and the results showed that cell growth inhibition by MALAT1 knockdown was partly reversed upon erastin treatment after cotransfection with the miR-145-5p inhibitor (Fig. 6A). Similarly, the results for intracellular Fe^2+^ and Liperfluo were reversed with miR-145-5p inhibition in MALAT1 knockdown cells under erastin treatment (Fig. 6B, C). Furthermore, knockdown of MALAT1 decreased the MUC1 protein level, while cotransfection with the miR-145-5p inhibitor partially restored MUC1 expression (Fig. 6D). Together, the above data demonstrated that silencing MALAT1 facilitated erastin-induced ferroptosis in EESCs through miR-145-5p/MUC1 signaling.

### Erastin-induced ferroptosis shrinks endometriotic lesions by regulating the MALAT1/miR-145-5p/MUC1 axis in vivo

**Fig. 7:**
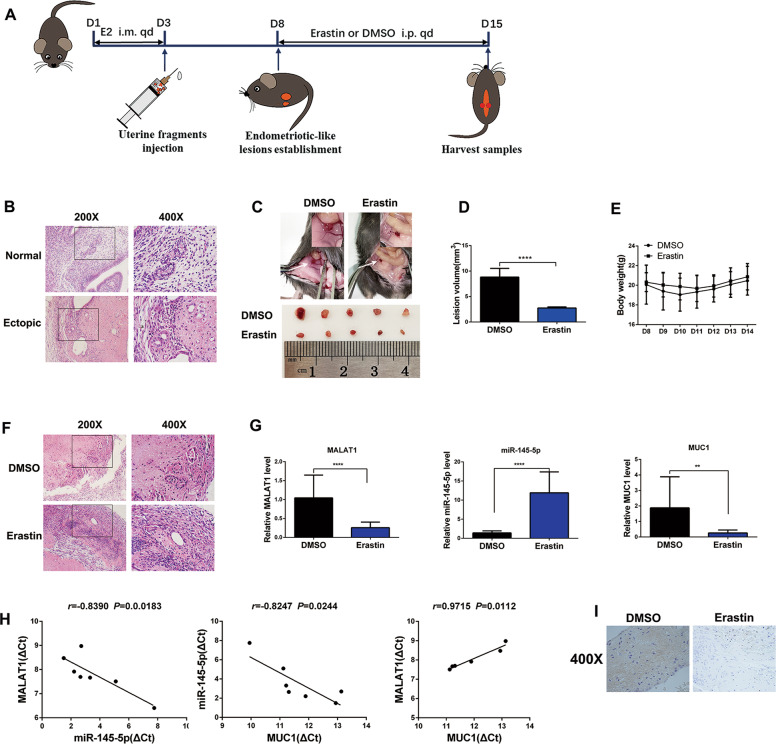
Erastin-induced ferroptosis shrinks endometriotic lesions by regulating the MALAT1/miR-145-5p/MUC1 axis in vivo. **A** Flowchart for the establishment of the mouse endometriosis therapeutic model. **B** The successful establishment of the endometriosis model was verified by H&E staining of ectopic lesions. The scale bar = 100 µm for ×200, 50 µm for ×400. **C** Representative visible lesions in the peritoneal cavity of a mouse model of endometriosis after treatment with erastin (20 mg/kg) or DMSO for 7 days are shown. **D** The volume of ectopic lesions was reduced after treatment with 20 mg/kg erastin for 7 days. **E** Body weights of the DMSO group and erastin group measured every day. Body weight did not change significantly after erastin treatment compared with the control group. **F** H&E staining showed that the glandular and stromal structures of ectopic lesions completely disappeared after erastin treatment. **G** The mRNA expression of MALAT1, miR-145-5p, and MUC1 in the DMSO group and erastin group was analyzed by qRT-PCR. **H** Correlations between the expression of MALAT1 and miR-145-5p, miR-145-5p and MUC1, MALAT1 and MUC1 in the erastin group were analyzed using the Pearson’s test. **I** The protein expression of MUC1 was detected by immunohistochemical staining. The scale bar =50 µm for ×400. All data are shown as the mean ± SD of three independent experiments (**P < 0.01, ****P < 0.0001, Student’s *t* test). MALAT1 metastasis-associated lung adenocarcinoma transcript 1, MUC1 mucin 1, i.p. intraperitoneal injection, i.m. muscle injection, E2 17-β-estradiol-3-benzoate, qod every other day, qd once a day.

To further explore whether erastin-induced ferroptosis inhibits the development of endometriosis in vivo, we established a mouse model of endometriosis. Endometriotic lesions were allowed to become established for 5 days before DMSO or erastin was injected i.p. (Fig. 7A). Next, hematoxylin and eosin (HE) staining showed the successful establishment of endometriotic-like lesions with stromal and epithelial cells (Fig. 7B). As shown in our therapeutic model, we observed that ectopic lesion volumes were reduced after treatment with 20 mg/kg erastin for 7 days (Fig. 7C, D). However, erastin had no effect on the body weight of mice (Fig. 7E). Meanwhile, HE staining showed that erastin destroyed the glandular and stromal structures of endometriotic lesions (Fig. 7F).

Furthermore, we observed that the expression levels of MALAT1 mRNA and MUC1 mRNA were decreased in the erastin-treated group compared with the DMSO group in vivo using qRT-PCR analysis. Meanwhile, the expression of miR-145-5p was found to be higher in the erastin-treated group than in the DMSO group (Fig. 7G). Pearson’s test showed that the MALAT1 mRNA level was negatively correlated with miR-145-5p mRNA but was positively correlated with the expression of MUC1 mRNA in the erastin treatment group. In addition, there was a negative correlation between the level of miR-145-5p mRNA and MUC1 mRNA in the erastin treatment group (Fig. 7H). Additionally, we found that the protein level of MUC1 was decreased after erastin treatment using an immunohistochemical staining assay (Fig. 7I). Collectively, these results indicate that erastin-induced ferroptosis shrinks endometriotic lesions by regulating the MALAT1/miR-145-5p/MUC1 axis in vivo.

## Discussion

In patients with endometriosis, according to the theory of retrograde menstruation, apoptotic endometrial tissue, cell debris, and erythrocytes reflux into the pelvic cavity. Lysed erythrocytes and released iron trigger oxidative stress by promoting the accumulation of ROS. Thus, EESCs live in an environment of iron overload and lipid peroxidation accumulation [23, 24]. Although both the current study and Li et al. indicated that ferroptosis resistance occurred in endometriosis [25], with the help of intrinsic iron and ROS overloading circumstances, EESCs were more sensitive to ferroptosis than NESCs [12]. In the present study, we confirmed that erastin triggers ferroptosis in EESCs.

Oxidative stress and lipid peroxidation are key events of ferroptosis [26, 27]. LncRNA MALAT1 promotes oxidative stress and ROS production in many diseases [28]. However, it has not been reported during ferroptosis. In the present study, we found that MALAT1 was downregulated during erastin-induced ferroptosis but was upregulated in ferroptosis-resistant EC tissues. Moreover, we found that knockdown of MALAT1 promoted cell growth inhibition of EESCs upon erastin treatment, and significantly increased the levels of intracellular iron, Liperfluo, and MDA in EESCs upon erastin treatment. These data suggested that knockdown of MALAT1 facilitated erastin-induced EESCs ferroptosis. The molecular mechanism by which MALAT1 is involved in erastin-induced ferroptosis in EESCs requires further exploration.

As predicted through a bioinformatic website, there existed a binding region between MALAT1 and miR-145-5p. Furthermore, this binding relationship between MALAT1 and miR-145-5p was confirmed using a dual-luciferase reporter assay. The above results indicated that miR-145-5p was a direct target of MALAT1. Notably, both the mRNA and protein levels of MUC1 were decreased by siMALAT1. MUC1, a suppressor of ferroptosis, has been repeatedly reported to be a direct target gene of miR-145 [21, 22, 29]. As expected, cell growth inhibition, intracellular Fe^2+^ levels, and the Liperfluo signal were rescued by MUC1 overexpression in cells transfected with miR-145-5p mimics under erastin treatment. In contrast, cell growth inhibition, and intracellular concentrations of Fe^2+^ and Liperfluo signal were reversed by MUC1 knockdown in cells transfected with miR-145-5p inhibitor under erastin treatment. These data indicated that miR-145-5p promoted erastin-induced ferroptosis in EESCs via MUC1. Furthermore, the results for the cell viability, intracellular Fe^2+^, and Liperfluo signal were reversed with miR-145-5p inhibition in MALAT1 knockdown cells under erastin treatment. In addition, we found that knockdown of MALAT1 decreased the expression level of MUC1 protein and was rescued to some extent after cotransfection of siMALAT1 and miR-145-5p inhibitor. The results indicate that silencing MALAT1 facilitates erastin-induced-ferroptosis in vitro through miR-145-5p/MUC1 signaling.

Finally, in a mouse model of endometriosis, erastin-induced ferroptosis shrunk the volumes of ectopic endometriotic lesions. Accordingly, the expression of MALAT1 mRNA and MUC1 mRNA was decreased, and the expression of miR-145-5p was increased in vivo after treatment with erastin. In addition, the MALAT1 mRNA level was negatively correlated with miR-145-5p mRNA but was positively correlated with the expression of MUC1 mRNA in the erastin treatment group. Moreover, the level of miR-145-5p mRNA was negatively correlated with MUC1 mRNA in the erastin treatment group. The above results indicate that erastin-induced-ferroptosis inhibited the development of endometriosis through the MALAT1/miR-145-5p/MUC1 axis in vivo.

In summary, knockdown of MALAT1 facilitates erastin-induced ferroptosis by targeting miR-145-5p/MUC1 signaling. The synergistic effect of MALAT1 knockdown and erastin induction in ferroptosis may be a new therapeutic strategy for endometriosis.

## Materials and methods

### Clinical samples

This study was approved by the Ethical Committee of the Second Affiliated Hospital of Harbin Medical University, and all patients provided informed written consent. The study recruited 25 women with endometriosis who were diagnosed by laparoscopy and histological analysis at the Second Affiliated Hospital of Harbin Medical University from March 2020 to April 2021. For the controls, normal endometriosis tissues were collected from 14 patients who underwent hysterectomy with uterine leiomyoma or grade II–III cervical intraepithelial neoplasia (CIN), and without clinical indication or history of endometriosis or adenomyosis.

### Primary endometrial stromal cells (ESCs) culture

Primary ectopic endometrial stromal cells (EESCs) and normal endometrial stromal cells (NESCs) were isolated according to a previously described method [30]. In brief, ectopic lesions from patients with ovarian endometrioma were collected and minced into 1 mm^2^ pieces. The minced tissues were digested with 4% collagenase type IV (C5138, Sigma, USA) for 60 min at 37 °C in a shaking water bath. Next, the dispersed endometrial cells were separated by 200 and 400 stainless steel mesh sieves. The filtrate was centrifuged at 1000 × *g* for 5 min. Then, the remaining cells were resuspended and cultured in DMEM containing 10% fetal bovine serum (FBS; Biological Industries, Israel). The ESCs were identified by immunofluorescent staining. Mycoplasma contamination was also tested.

### Cell transfection

The miR-145-5p mimics, miR-145-5p inhibitor, siRNA against MALAT1 and MUC1, MUC1 plasmid, and their respective negative controls were obtained from RiboBio (Guangzhou, China). Transfections were performed using Lipofectamine 3000 (Invitrogen, Carlsbad, USA) according to the manufacturer’s instructions. The target sequence of siRNA was as follows: siMALAT1, 5ʹ-CACAGGGAAAGCGAGTGGTTGGTAA-3ʹ. The corresponding siMALAT1 knockdown efficiency is shown in Supplementary Fig. 1A–C.

### RNA extraction and quantitative real-time PCR

Total RNA was extracted from frozen tissues or primary cultured cells using TRIzol reagent (Invitrogen, Carlsbad, USA), and 1 μg of total RNA was used for first-strand cDNA synthesis using a Reverse Transcription Kit (Toyobo Co, Osaka, Japan). For miRNA, RNA was reverse transcribed using Bulge-Loop miRNA-specific RT primers (RiboBio, Guangzhou, China). The quantitative real-time PCR (qPCR) protocol was performed using SYBR Green PCR Master Mix (Bio-Rad, Hercules, CA, USA) and an Applied Biosystems 7300 Real-Time PCR System. The sequences of primers used for qRT-PCR analysis are shown in Table 1. The expression levels of different genes were normalized using small nuclear RNA (snRNA) U6 or ACTB as an internal control.

**Table 1:** Sequences of primers used for qRT-PCR analysis.

Gene	Forward primer sequence	Reverse primer sequence
ACTB	AGCGAGCATCCCCCAAAGTT	AGGGCACGAAGGCTCATCATT
U6	CTCGCTTCGGCAGCACA	AACGCTTCACGAATTTGCGT
ACSL4	TTCGATTAAGCCCAGAGCCA	GAGGTAATGGTTCCTCAGCTCCT
GPX4	GGCAGACCCGAAAATCCAG	GTTTATTCCCACAAGGTAGCCA
SLC7A11	CCCTTTGCTCTCATACCCATC	GACTTTCCTCTTCAGCTGCACTT
LncRNA MALAT1	GAGATGAGTGGGATCGAGCG	GAAACCTGTCTGAGGCAAACG

*ACSL4* acyl-CoA synthetase long-chain family member 4, *GPX4* glutathione peroxidase 4, *SLC7A11* solute carrier family 7 member 11, *MUC1* mucin 1, *LncRNA MALAT1* long noncoding RNA metastasis-associated lung adenocarcinoma transcript 1, *qRT-PCR* reverse transcription and quantitative real-time PCR.

### Cell viability

Cell Counting Kit-8 (CCK-8) (MCE, Shanghai, China) was used to test cell viability according to the manufacturer’s instructions. Then, 100 µl of serum-free medium containing 10 µl of Cell Counting Kit-8 solution was added to the dishes and incubated for 1 h at 37 °C. Absorbance at 450 nm was measured using a plate reader (Bio-Rad).

### Measurement of intracellular Fe^2+^ amount

FerroOrange (Dojindo, Kumamoto, Japan) was used to detect the intracellular Fe^2+^ amount according to the manufacturer’s instructions. In the confocal dish, cells were incubated with 1 µM FerroOrange in HBSS for 30 min at 37 °C. Fluorescence images were obtained using a Nikon Confocal C2 fluorescence microscope from 3 separate dishes for each treatment.

### Calcein-AM/PI fluorescence staining

A Calcein-AM/PI Double Stain Kit (Solarbio, Beijing, China) was used according to the manufacturer’s instructions. Calcein-AM (2 μmol/l) and propidium iodide (4.5 μmol/l) were added to the culture wells and incubated at 37 °C in the dark for 15 min. Living cells (green cytoplasmic fluorescence) and dead cells (red nucleus) were observed with an inverted fluorescence microscope (Nikon, Tokyo, Japan).

### Ferroptosis detection by Liperfluo

Lipid peroxidation was detected via Liperfluo (Dojindo, Kumamoto, Japan). Cells were incubated with 5 μM Liperfluo for 30 min at 37 °C in accordance with the manufacturer’s instructions. After removing Liperfluo, EESCs were washed three times with HBSS. Fluorescence microscopy (Nikon, Tokyo, Japan) was used to obtain fluorescence images from 3 separate dishes for each treatment.

### Measurement of MDA

An MDA assay kit (Beyotime, Jiangsu, China) was used to measure the intracellular levels of MDA. The treated EESCs were collected and the content of intracellular MDA was determined according to the instructions provided by the kit.

### Dual-luciferase reporter assay

For luciferase reporter assays, the putative miR-145 binding sites on MALAT1 RNA were cloned downstream of the cytomegalovirus (CMV) promoter in the pMIR-REPORT vector (Ambion, Carlsbad, CA, USA). Luciferase activity was measured using a Dual-Luciferase Reporter Assay System (Promega, Madison, WI, USA) according to the manufacturer’s instructions. Briefly, pMIR-REPORT-MALAT1 or pMIR-REPORT-MALAT1-mut was cotransfected with the miR-145 mimics or mimic NC into 293T cells using Lipofectamine 2000 according to the manufacturer’s instructions. Luciferase activity was normalized to Renilla luciferase activity at 48 h after transfection.

### Western blot (WB)

WB was conducted according to conventional protocols. Briefly, protein from cells was extracted with RIPA buffer (Beyotime, China). Proteins were loaded in SDS-PAGE gels and transferred to PVDF membranes (Millipore, USA). After blocking, membranes were incubated with primary antibodies (Table 2) overnight at 4 °C, and secondary antibodies for 2 h at room temperature. Membranes were exposed and developed after immersion in ECL reagent (Epizyme, China).

**Table 2:** Details of antigens used in WB assays.

Antigen	Catalog number	Dilution	Source	Species
ACSL4	22401-1-AP	1:200	Proteintech	Rabbit
GPX4	67763-1-Ig	1:2000	Proteintech	Mouse
SLC7A11	26864-1-AP	1:500	Proteintech	Mouse
MUC1	23614-1-AP	1:200	Proteintech	Rabbit

*WB* western blot, *ACSL4* acyl-CoA synthetase long-chain family member 4, *GPX4* glutathione peroxidase 4, *SLC7A11* solute carrier family 7 member 11, *MUC1* mucin 1.

### Immunohistochemistry staining

Immunohistochemistry staining assays were conducted according to conventional protocols. Briefly, processed specimens were cut into 4-µm sections. The sections were incubated with primary antibodies (Table 3) for 16 h at 4 °C and then incubated with secondary antibodies for 30 min at room temperature. Then all sections were incubated with diaminobenzidine (DAB)-substrate (CWBIO, Beijing, China) to develop positive staining and counterstained with hematoxylin to detect HRP activity.

**Table 3:** Details of antigens used in immunohistochemistry analyses of EN and EC tissues.

Antigen	Catalog number	Dilution	Source	Species
ACSL4	22401-1-AP	1:50	Proteintech	Rabbit
GPX4	67763-1-Ig	1:1000	Proteintech	Mouse
SLC7A11	26864-1-AP	1:200	Proteintech	Mouse
MUC1	23614-1-AP	1:50	Proteintech	Rabbit

*WB* western blot, *ACSL4* acyl-CoA synthetase long-chain family member 4, *GPX4* glutathione peroxidase 4, *SLC7A11* solute carrier family 7 member 11, *MUC1* mucin 1.

### Establishment of the endometriosis mouse model

All animal handling and experimental procedures were approved by the Animal Experimental Ethics Committee of Harbin Medical University. A mouse model of endometriosis was established as previously described [30]. Seven-to-8-week-old C57BL/6 female mice were obtained and 17-β-estradiol-3-benzoate (30 µg/kg, Sigma) was administered to each mouse every day for 3 days. We removed uterine horns from the donor mice and added them to saline. Endometrium was cut into 1 mm^2^ fragments. The endometrial fragments from each uterine horn were suspended in 0.3 ml saline and injected into the peritoneal cavities of recipient mice with an 18-gauge needle. At 8 days (5 days after the operation), endometrial-like lesions were established, and they were randomly divided into two groups (each group contained 12 mice). In the experimental group, each mouse received erastin (20 mg/kg/day) by intraperitoneal injection over a 7-day period. In the control group, DMSO was used instead of erastin. At 15 days, the mice were sacrificed and endometriotic lesions were collected (Fig. 7A). The length and width of ectopic lesions were measured and the volumes of lesions were calculated by the prolate ellipsoid geometric model: 1/2 (length × width^2^).

### Statistical analysis

The statistical analyses in this study were carried out using Prism 7. The experimental data are presented as the mean ± standard deviation (SD) from at least three independent experiments. All statistical analyses were performed using Student’s *t* test. Differences were considered significant at *P* < 0.05. NS is nonsignificant (*P* ≥ 0.05), *P < 0.05, **P < 0.01, ***P < 0.001, ****P < 0.0001.

## Supplementary information

Determination of siMALAT1 transfection efficiency.

Figure legend of Supplementary Fig.1

The full length uncropped original western blots.

## Data Availability

The original contributions presented in the study are included in the article/Supplementary Material, further inquiries can be directed to the corresponding author.
