# Supplementary figures and images for: Silencing of lncRNA MALAT1 facilitates erastin-induced ferroptosis in endometriosis through miR-145-5p/MUC1 signaling
Source: Cell Death Discov. 2022 Apr 11;8:190. doi: 10.1038/s41420-022-00975-w (PMC8995383; doi:10.1038/s41420-022-00975-w)

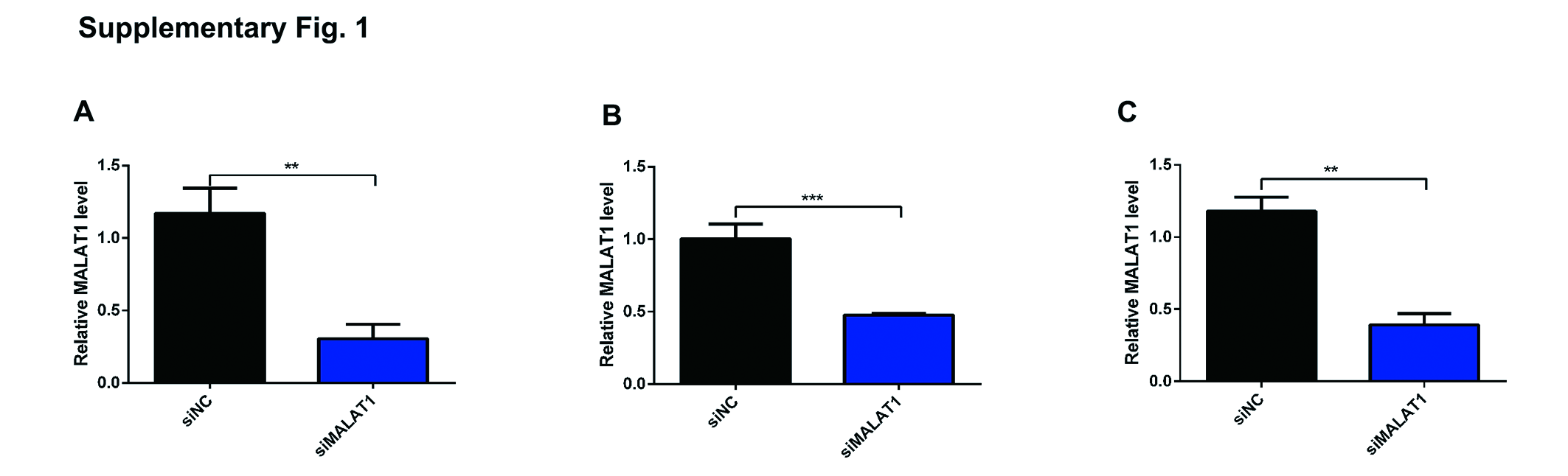

Supplement: Supplementary file 1 — Determination of siMALAT1 transfection efficiency. [file 41420_2022_975_MOESM1_ESM.tif]

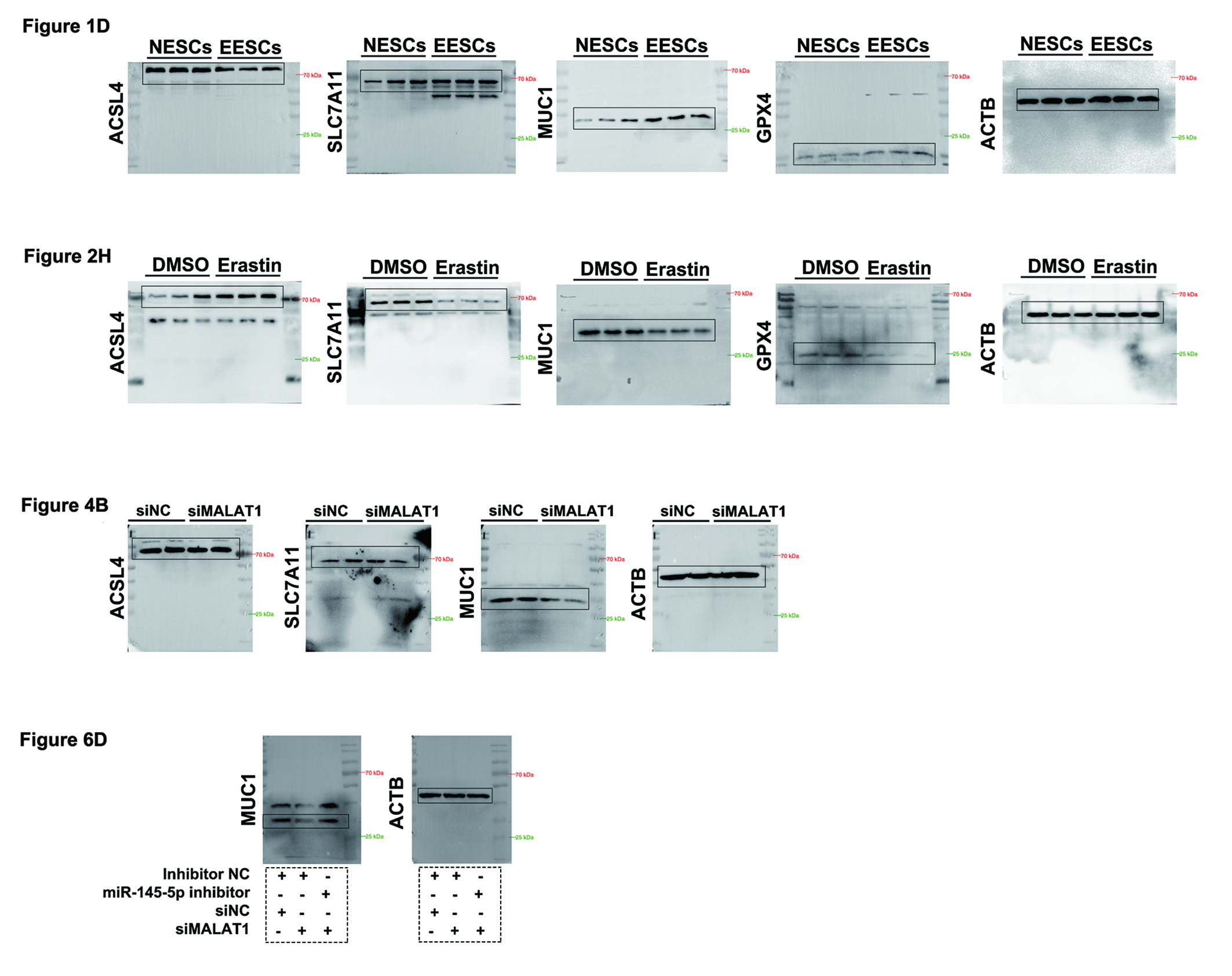

Supplement: Supplementary file 3 — The full length uncropped original western blots. [file 41420_2022_975_MOESM3_ESM.tif]
