# Supplementary material for: Silencing of lncRNA MALAT1 facilitates erastin-induced ferroptosis in endometriosis through miR-145-5p/MUC1 signaling
Source: Cell Death Discov. 2022 Apr 11;8:190. doi: 10.1038/s41420-022-00975-w (PMC8995383; doi:10.1038/s41420-022-00975-w)
Supplement: Supplementary file 2 — Figure legend of Supplementary Fig.1 [file 41420_2022_975_MOESM2_ESM.docx]

**Supplementary Fig. 1 Determination of siMALAT1 transfection efficiency.** The transfection efficiency of siMALAT1 was detected by qRT-PCR assays in parallel with WB assays（Supplementary Fig. 1A）, MDA assays (Supplementary Fig. 1B), CCK8, FerroOrange, and Liperfluo assays (Supplementary Fig. 1C).
